# *Leptin* Genes in Blunt Snout Bream: Cloning, Phylogeny and Expression Correlated to Gonads Development

**DOI:** 10.3390/ijms161126044

**Published:** 2015-11-18

**Authors:** Honghao Zhao, Cong Zeng, Shaokui Yi, Shiming Wan, Boxiang Chen, Zexia Gao

**Affiliations:** 1 College of Fisheries, Key Lab of Agricultural Animal Genetics, Breeding and Reproduction of Ministry of Education/Key Lab of Freshwater Animal Breeding, Ministry of Agriculture, Huazhong Agricultural University, Wuhan 430070, China; zhao253091640@hotmail.com (H.Z.); Congzeng@live.cn (C.Z.); yishaokui@foxmail.com (S.Y.); wanshiming@webmail.hzau.edu.cn (S.W.); ChenBX@haid.com.cn (B.C.); 2 Freshwater Aquaculture Collaborative Innovation Center of Hubei Province, Wuhan 430070, China; 3 Hubei Bai Rong Improved Aquatic Seed Co., Ltd., Huanggang 438800, China

**Keywords:** *Megalobrama amblycephala*, *leptin* genes, cloning, gene expression, gonad development

## Abstract

To investigate the leptin related genes expression patterns and their possible function during the gonadal development in fish, the cDNA and genomic sequences of *leptin*, leptin receptor (*leptinR*), and leptin receptor overlapping transcript like-1 (*leprotl1*) were cloned and their expression levels were quantified in the different gonadal development stages of *Megalobrama amblycephala*. The results showed that the full length cDNA sequences of *leptin*, *leptinR* and *leprotl1* were 953, 3432 and 1676 bp, coding 168, 1082, and 131 amino acid polypeptides, and the genomic sequences were 1836, 28,528 and 5480 bp, which respectively had 3, 15 and 4 exons, respectively. The phylogenetic analysis revealed that three genes were relatively conserved in fish species. Quantitative real-time PCR results showed that the three genes were ubiquitously expressed in all examined tissues during the different gonadal development stages. The *leptin* and *leptinR* took part in the onset of puberty, especially in female *M. amblycephala*, by increasing the expression levels in brain during the stage I to III of ovary. The expression levels of *leptin* and *leptinR* had significant differences between male and female in hypothalamic-pituitary-gonadal (HPG) axis tissues (*p* < 0.05). The *leptinR* had the same variation tendency with *leptin*, but the opposite changes of expression levels were found in *leprotl1*, which may resist the expression of *leptinR* for inhibiting the function of leptin in target organ. These findings revealed details about the possible role of these genes in regulating gonadal maturation in fish species.

## 1. Introduction

Leptin, a hormone mainly secreted by fat, is the protein product of the obese gene (*OB*) and belongs to the type I cytokine family. Since Zhang [1] successfully cloned *OB* gene in mice by positional cloning technologies, many studies had been conducted on Leptin orthologous [2,3]. As to teleosts, *leptin* gene was firstly characterized in pufferfish (*Takifugu rubripes*) [4]. Subsequent identifications of *leptin* had been reported in other fish species, including goldfish (*Carassius auratus*) [5], common carp (*Cyprinus carpio*) [6], rainbow trout (*Oncorhynchus mykiss*) [7], zebrafish (*Danio rerio*) [8], grass carp (*Ctenopharyngodon idella*), silver carp (*Hypophthalmichthys molitrix*) [9], and yellow catfish (*Pelteobagrus fulvidraco*) [10]. Leptin receptor (*leptinR*) could mediate the physiological actions of leptin through associating with membrane, so it is necessary to analyze the *leptinR* to know the physiological role of leptin in fish. Right now, leptinR of many fish species have been identified, including zebrafish (*D. rerio*) [11], puffer fish (*T. rubripes*) [12], Japanese medaka (*Oryzias latipes*) [13] and crucian carp (*C. auratus*) [14]. As to leptin receptor overlapping transcript gene (*leprot*), it shares the first and second exons with *leptinR* in human, and had been suggested that both *leptinR* and *leprot* were under control of a single promoter and encoded by a single gene [15]. However, Kurokawa *et al.* [12] suggested that *leptinR* and *leprotl* of pufferfish were homologous genes with human *leptinR* and *leprotl*, but pufferfish *leptinR* and *leprotl* were not located sequentially on the same chromosome. Kurokawa and Murashita [13] also found that leptinR and leprotl were encoded by separate genes in medaka (*Oryzias latipes*). Generally, the information about fish *leprotl* was relatively scanty.

Although the function of *leptin* has been explored extensively in mammals, its role in fish is far from fully understood. Most studies about the physiological role of *leptin* had been focused on its function in appetite [16], body weight regulation [5] and metabolism [17] in teleosts. As in many mammalian species, the nutritional status of an individual is important for initiation of sexual maturation in teleosts [18]. However, few researches had considered the possible role of leptin in teleost reproduction [18,19]. In a previous study, Peyon [20] demonstrated that recombinant mouse leptin stimulated basal release of luteinizing hormone (LH) in sea bass (*Dicentrarchus labrax*) pituitary cell culture in the late pre-pubertal, early post-pubertal and adult stages. In an *in vitro* study on female rainbow trout (*Oncorhynchus mykiss*), leptin protein, directly stimulated follicle-stimulating hormone (FSH) and LH release by acting at the level of the pituitary [21]. Trombley and Schmitz [18] had proved that *leptin* had a possible physiological role during sexual maturation in male Atlantic salmon (*Salmo salar*). As to the *leptinR* and *leprotl1*, few studies have been conducted to investigate their possible role in fish maturation and expression correlation with *leptin* gene.

Blunt snout bream (*Megalobrama amblycephala*) is naturally habited in inland lakes along the Yangtze River. Due to its desirable qualities for aquaculture such as herbivorous feeding habit, general hardiness, resistance to disease, good seinability and reproductive performance, *M. amblycephala* has been recognized as one of the main freshwater aquaculture species since the 1960s in China. Recently, its total production is enormously and vigorously growing [22]. However, after domestication of blunt snout bream since the 1960s, germplasm resources of this bream are under threat of recession and mixture due to its artificial breeding. In a cultured *M. amblycephala* population, early sexual maturity was found at one year old, which was normally reached by two or three years of age in natural populations [23]. In this study, we isolated and characterized the *leptin*, *leptinR* and *leprotl1* genes of *M. amblycephala* and detected their expression patterns during different gonadal developmental stages to investigate the relationships between these three genes and fish maturation.

## 2. Results

### 2.1. Identification and Characterization of Three Genes

**Figure 1:**
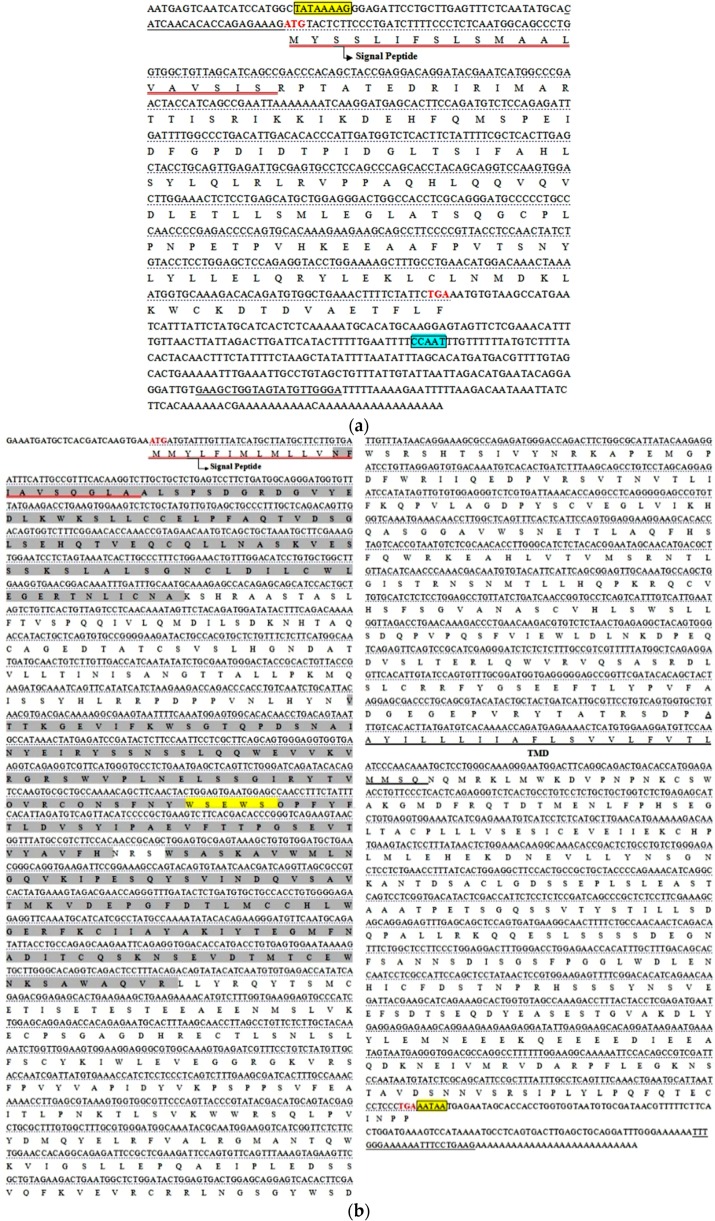

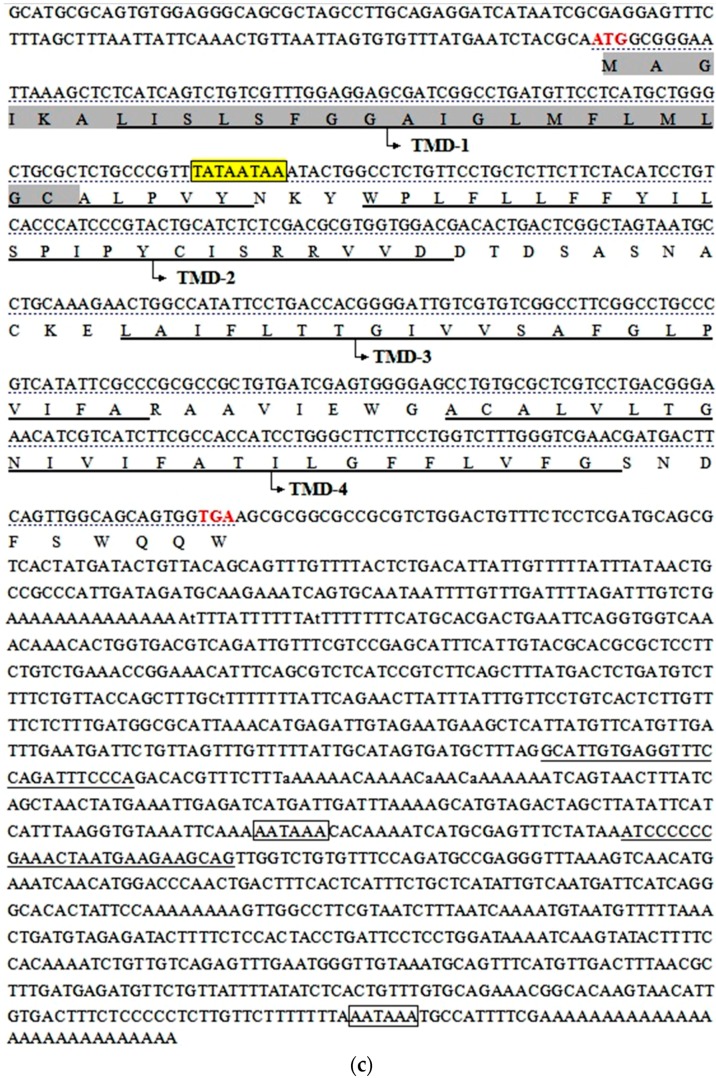
Nucleotide and deduced amino acid sequences of *M. amblycephala leptin* (**a**); *leptinR* (**b**); and *leprotl1* (**c**). The open reading frame (ORF) were underlined; the start and stop codons were bold and red; the signal peptide sequence were red underlined and marked; the transmembrane domains were underlined and marked with transmembrane domain (TMD); the conserved site sequences were boxed and shaded; the deduced amino acid sequences of fibronectin type-III domain profiles of *leptinR* were filled in gray and a conserved motif (WSXWS) in *leptinR* was boxed and shaded; predicted amino acid sequence of prokaryotic membrane lipoprotein lipid attachment site profiles in *leprotl1* were filled in gray. The TATA boxes and conservative motif have been highlighted in yellow, the transcription factor “CCAAT” was marked in blue.

**Figure 2:**
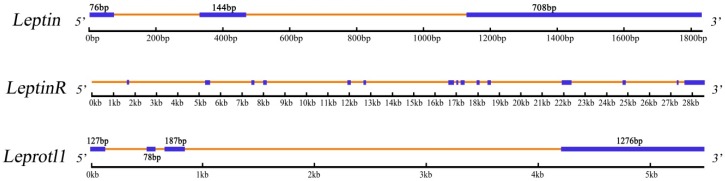
The whole genome of *M. amblycephala leptin*, *leptinR* and *leprotl1* were shown with predicted coding exons and introns with their length. The exons were represented by blue threads and introns were represented by yellow threads.

The full length cDNA sequences of *leptin, leptinR,* and *leprotl1* of *M. amblycephala* were 953, 3432 and 1676 bp, coding 168, 1082, and 131 amino acid polypeptides, respectively. The genomic sequences of *leptin, leptinR,* and *leprotl1* genes were 1836, 28,528 and 5480 bp, which respectively had 3, 15 and 4 exons. The basic characterizations of these three genes were showed in Table 1. The regulating elements of TATA box and C/EBP binding sequence were found from the upstream 52 bp of the promoter in *leptin* 5′ flanking region. In *leptinR*, there was a splice site at 18th aa. The conserved structures and the basic characteristics of the three genes were showed in Figure 1. The exon and intron domains were identified by comparing the full-length cDNA sequences with their whole genomic sequences, and the results were showed in Figure 2.

**Table 1:** The basic characterizations of three genes in *M. amblycephala*.

Target Gene	*Leptin*	*LeptinR*	*Leprotl1*
cDNA length	953	3432	1676
5’-Untranslated Region (UTR)	81	25	111
Open Reading Frame (ORF)	514	3249	396
ORF Encoding aa	168	1082	131
3’-UTR	371	158	1169
GeneBank No.	KJ 193854	KJ 193855	KJ 193853
CpG Island	0	4	1
Transmembrane Domain (TMD)	0	1	4
Signal Peptide	1	1	0
Fibronectin type-III domain	0	3	0
Genome Length (bp)	1836	28,528	5480
Exon	3	15	4
GeneBank No.	KP 269244	KP 269245	KP 269246

### 2.2. Phylogenetic Evolution Analysis

**Figure 3:**
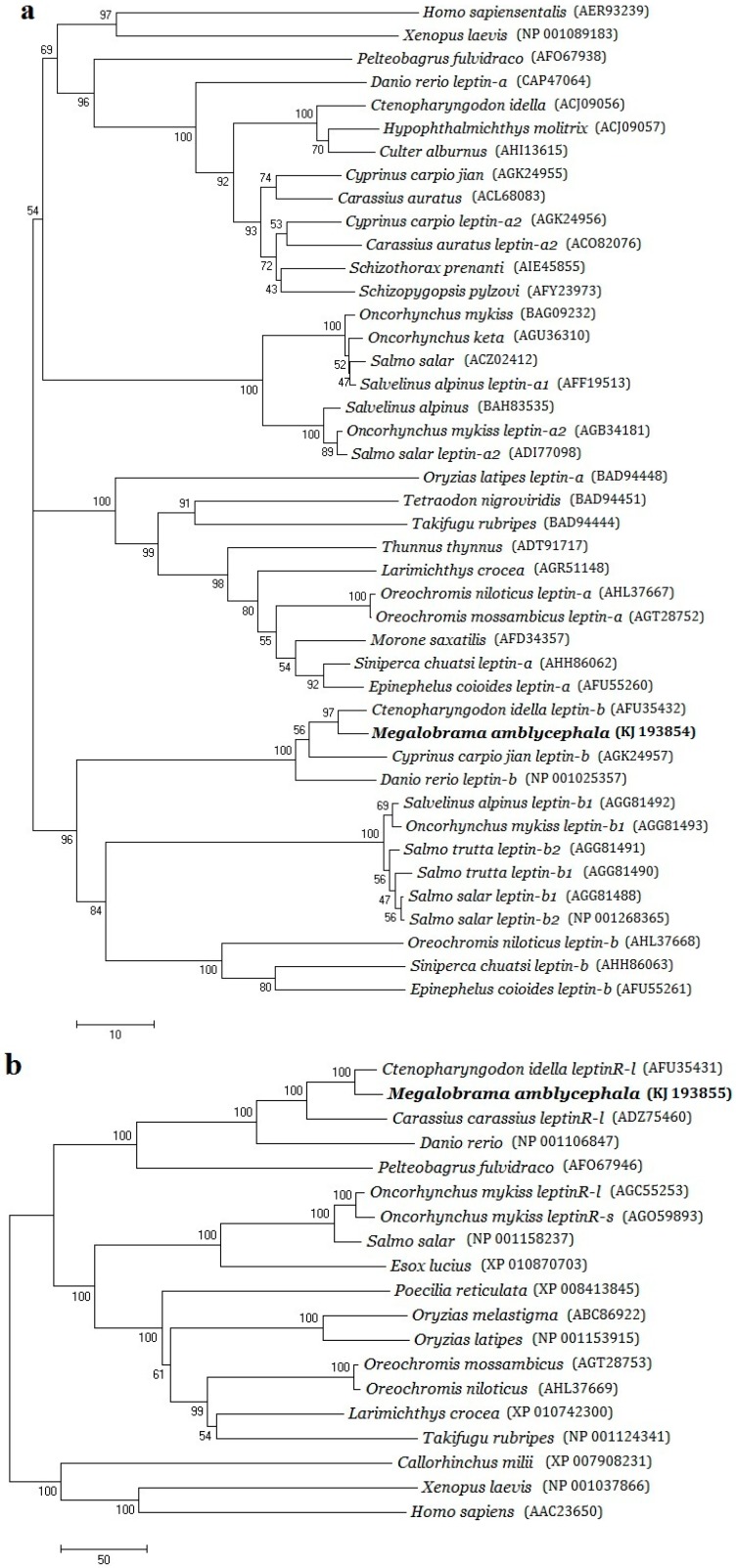

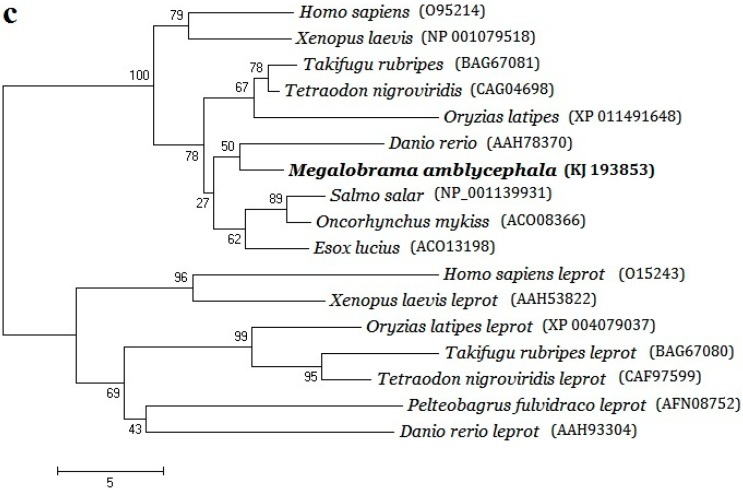
Phylogenetic tree of *M. amblycephala* *leptin* (**a**); *leptinR* (**b**); and *leprotl1* (**c**). Phylogenetic tree was constructed by neighbor-joining method in Clustal X and MEGA 5.0 program by alignment of the ORF encoding peptide sequences of *Leptin* gene family among *M. amblycephala* and other organism species. The GenBank accession No. was also showed behind the Latin name of each individual.

Phylogenetic trees of the three genes are shown in Figure 3, and the multiple amino acid sequences alignments of *leptin*, *leptinR* and *leprotl1* are supplied as Supplemental Figures S1–S3. The consequences of multiple amino acid sequences alignment revealed that the conservative amino acid fragments of *M. amblycephala leptin* were malposed by compared to Perciformes, Tetraodontiformes and Cyprinodontiformes, which were also separated in different branches of the *leptin* phylogenetic tree. Phylogenetic tree of *leptin* had two branches and the obtained *M. amblycephala leptin* formed a cluster with fishes *leptin*-B (Figure 3A).

The multiple amino acid sequences alignment of *leptinR* showed that *M. amblycephala leptinR* shared the highest similarities with *C. idella* and the WSEWS motif that located in the second fibronectin type-III domain profile shared in all compared species (Figure 3B). In phylogenetic tree, *M. amblycephala leptinR* gathered in one branch with Cypriniformes and Siluriformes long-form *leptinR*. This result was in accordance with the conventional taxonomic relationship of these species.

The predicted amino acid sequence of *M. amblycephala leprotl1* included a prokaryotic membrane lipoprotein lipid attachment site and three transmembrane domains, which were shared in all compared species. The phylogenetic analysis of *leprotl1* (Figure 3C) showed that *M. amblycephala* was relatively more close to *D. rerio*, *S. salar* and *O. mykiss* than the other teleostean species.

### 2.3. Expression Analysis in Different Developmental Stages of Gonads

**Figure 4:**
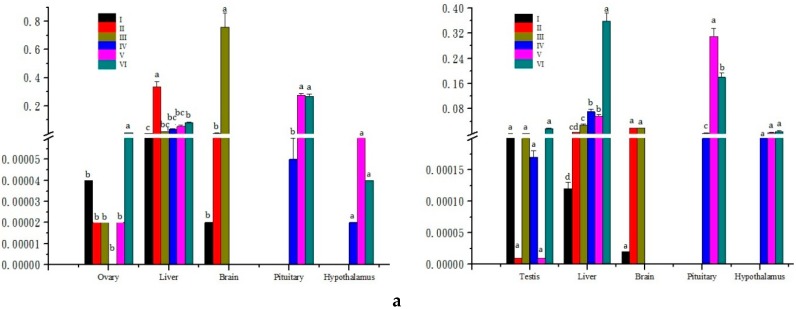

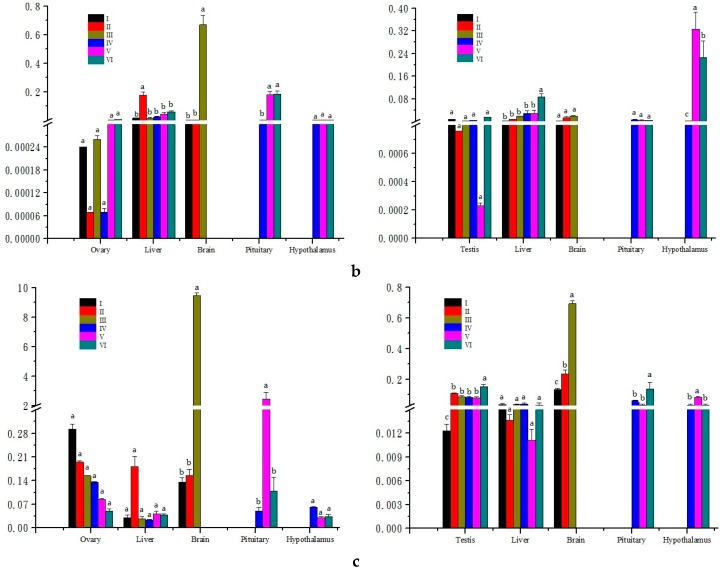
The expression levels of *M. amblycephala leptin* (**a**); *leptinR* (**b**); and *leprotl1* (**c**) in different tissues during the developmental periods of ovary (**left**) and testis (**right**). The β-*actin* was used as an internal control to calibrate the cDNA template for all the samples. Vertical bars represent the mean ± SE, and bars with different letters mean significantly different (*p* < 0.05).

The results of mRNA expression quantity of three genes in the five examined tissues during the different developmental stages of gonad were showed in Figure 4. No significant variation was observed in the expression levels of *leptin* in testis except the stage VI which was the highest; while *leptin* showed differential expression with relatively lower levels in ovary compared to testis (*p* < 0.05), and the highest expression level was also detected at stage VI of ovary. The expression levels in liver of the *leptin* gene were highest at stage II of female and stage VI of male, with relatively higher expression quantity in male compared to female from stage III to VI. Moreover, the expression of *leptin* in pituitary increased to the peak from stage IV to V and then again decreased at stage VI of both male and female, respectively. The variation tendency of expression level in *M. amblycephala* female hypothalamus was totally different from the male. For the female, the levels increased to the peak value from stage IV to V and then decreased at stage VI; but for the male, the levels increased to the peak value at stage VI.

Significant variation was observed in the expression levels of *leptinR*, whereas male and female showed differential expression in the same tissue at the same stage (*p* < 0.05). The expression levels of *leptinR* were highest at stage VI for both ovary and testis with different variation tendency. For female, the levels in the ovary were increased from stage II to III and decreased at stage IV, then increased again to the peak value at stage VI; the levels in the testis were in fluctuation, respectively. In addition, the expression levels of *leptinR* in both male and female hypothalamus increased to the peak value from stage IV to V and then decreased at stage VI. The variation tendency of expression quantity in *M. amblycephala* female pituitary was totally different from the male. For female, the levels increased to the peak value from stage IV to VI, while the levels in male decreased from stage IV to stage VI and the highest level was detected at stage IV. In addition, the significant differences of expression levels for *leptinR* between males and females were showed in Supplemental Table S1 (*p* < 0.05).

Finally, *M. amblycephala leprotl1* had different expression patterns from both *leptin* and *leptinR*. At first, the expression levels of *leprotl1* in ovary were decreased during the different developmental stages, while the levels in testis were increased from stage I to stage II and then decreased from stage II to stage V; at last, the levels increased to the peak value at stage VI. Meanwhile, the variation trend of expression quantity of *leprotl1* in brain was the same as *leptin* and *leptinR*. The expression levels of *leprotl1* also had significant differences between male and female (*p* < 0.05) (Supplemental Table S1).

### 2.4. Correlation Analysis of Three Genes

The correlation coefficients among *leptin*, *leptinR* and *leprotl1* mRNA expression in the different development stages of *M. amblycephala* gonad were showed in Table 2. The results of correlation analysis showed that there were significant positive correlations between every two genes (*p* < 0.01) and the three genes (*leptin*, *leptinR* and *leprotl1*) all had fairly strict relevance with the other two (*r* > 0.7).

**Table 2:** The correlation coefficients among *leptin*, *leptinR* and *leprotl1* expression in the different development stages of *M. amblycephala* gonad.

Target Gene	*Leptin*	*Leptinr*	*Leprotl1*
*leptin*	–	0.752 **	0.751 **
*leptinR*	0.000	–	0.799 **
*leprotl1*	0.000	0.000	–

Data above the diagonal showed the development-related correlation coefficient and the below expressed the level of significant difference. **, correlation is significant at the 0.01 level (2-tailed).

## 3. Discussion

### 3.1. Structural Characterizations and Evolution Analysis of Three Genes

The *leptin*, *leptinR* and *leprotl1* of *M. amblycephala* were isolated, cloned and characterized for *M. amblycephala* in the present study. The functionally important residues have been predicted, while the results showed that some functional domains in *M. amblycephala leptin*, *leptinR* and *leprotl1* sequences were highly conserved among fish species. These three genes are highly conserved molecular chaperones among the course of evolution in teleost [24]. In addition, many conservative amino acid residues were discretely existed in the sequences of the teleosteans, amphibians, birds, mammalians and human *leptin*s, which may be caused by the base deletion, gene mutation or rearrangement that occurred in the process of gene replication and many other mechanisms of adaptive evolution, meanwhile these heritable variations were reserved during the species evolution [25].

In fish, prior studies also proposed that all fishes expressed two *leptin* paralogs [8], with the possible exception of *T. rubripes* [4], while *M. amblycephala leptin* took the same branch with Cyprinid, Salmonidae, Bagridae fishes *leptin-B* and kept away from other vertebrates *leptin-A*, therefore we deduced that *M. amblycephala leptin* should belong to the type B. Meanwhile our *leptin* was in disparate branch with the *C. idella,* which had closest ties of consanguinity with *M. amblycephala*. This result could be due to one or more *leptin-B* paralogs being in existence [26]. On the other hand, more recent work indicated that some fishes lost the second *leptin-B* ortholog, such as striped bass *Morone saxatilis*, stickleback *Gasterosteus aculeatus* [16] and Chinese perch *Siniperca chuatsi* [27]. The leptin of *M. amblycephala* entirely belonged to the type B, whether *M. amblycephala leptin* has the type A needs to be further studied.

Moreover, *M. amblycephala leptinR* had nearest genetic distance with *C. idella*, followed by *C. auratus*, *C. carpio* “*jian*” and *D. rerio* respectively*.* These results were in accordance with conventional taxonomy of teleosts, similar results were found in *GHR*s, *IGF*s and *MSTN*s genes of *M. amblycephala* [28]. Additionally, two forms of *leptinR* have been identified in previous studies. The most obvious grouping of *leptinR* forms is to distinguish the length of intracellular domain and the *leptinR*-long is the signaling transduction form [29]. By compared with other species *leptinR*s, *M. amblycephala leptinR* should be the long-form.

*M. amblycephala leprotl1* occupied a branch with *L. oculatus*, *L. chalumnae*, *S. salar*, *O. mykiss*, *T. nigroviridis*, *O. niloticus* and *Xenopus laevis*. It had relatively higher homologies with *L. chalumnae*, *O. niloticus* and *T. nigroviridis*. However, due to the information involved in *leprotl* gene was very limited in fish, we cannot ensure the evolution state of *M. amblycephala leprotl1*.

In general, the three genes were conserved in population evolution due to their important functions [30]. The results of adaptive evolution indicated that the branch of these *M. amblycephala* genes had undergone Darwin positive selection to adapt ever-changing environment [24,31].

### 3.2. Analysis of Genes Exons and Introns

In the present study, the regulating elements of TATA box and C/EBP binding sequence were found from the upstream 52 bp of the promoter in *M. amblycephala leptin* 5′ flanking region. The mutation experiment found that the TATA box and C/EBP binding sequence had significant effects on the expression of mouse *leptin* [32], as the point mutation of various regulatory elements would cause the decline of promoter activity. If multiple regulatory elements mutated at one time, it would produce the superimposed effect. Therefore *M. amblycephala leptin* sequence had multiple regulatory sites. The functions of the regulating elements in 5′ flanking region for regulating the transcription of *M. amblycephala* leptin needs future studies.

By comparing and aligning the genomic, transcript or protein sequences, the evolution of genes was studied and reconstructed [25]. Fishes, by far, were the logical organisms to study *leptin* gene among non-mammal species [33]. In contrast to the whole genome structure of other vertebrates *leptin* [27], in present study, we found that *M. amblycephala leptin* gene was encoded by 3 exons, they were separated by a 252 bp intron with consensus 5′ donor (GT) and 3′ acceptor (AG) splice sites and a 660 bp intron, respectively. The number of *leptin* intron and exon was unanimous by comparing the *C. idella*, *H. molitrix*, human and mice *leptin*s [34]. For the structure of teleostean *leptin*s, the length of the intron1, 2 and exon3 had significant discrepancy among species.

There are some studies have indicated that *leptinR* and *leprot* located in the same chromosome, which transcripts shared the first two exons that were not translated in the *leptinR* gene [35]. In our study, *M. amblycephala leptinR* and *leprotl1* didn’t share the first two exons. However, the genome references of *M. amblycephala* are not available, so we couldn’t define whether *M. amblycephala leptinR* locate in the same chromosome with *leprotl1*.

### 3.3. Expression Analysis of Three Genes

According to the results of quantitative analysis of three genes in relative tissues of HPG axis at different developmental stages of gonad, we found that this axis played a major role in the regulation of gonadal development and functions. In addition to the simulative effect at the hypothalamus level, leptin had direct actions on the anterior pituitary [36]. Gonadotropin-releasing hormone (GnRH) was released by hypothalamus neurons, and then it could stimulate the release of pituitary gonadotropin, including luteinizing hormone (LH) and follicle-stimulating hormone (FSH), which regulate the formation of sex hormones and germ cell, respectively [37]. The leptin may indirectly regulate the development of *M. amblycephala* gonad and took part in the reproductive manipulation through the HPG axis [38]. The leptin may act as an important factor to link between the reproductive system and adipose tissue, which could indicate whether adequate energy are available for normal reproductive action [39]. The leprotl negatively regulates cell surface expression of growth hormone (GH) receptor in liver. It may participate in resistance to GH in liver during stages of reduced available nutrient [40]. In present study, we can also speculate that leprotl1 may negatively regulated surface expression of leptinR, which may participate in resisting to leptin.

The increasing expression levels of *leptin* and *leptinR* genes were detected at stage III in brain and the booming expression levels in liver at stage II declared that *M. amblycephala* stimulated energy in liver at stage II for *Leptin* gene system and indirectively participated in the reproductive start of *M. amblycephala* at stage III by brain physiological signals. The increasing levels of *leptinR* in gonad explained that gonad was the main target organ of reproductive start, while the testis had the same variation tendency with ovary. The expression levels of *Leptin* gene system had significant differences between males and females in HPG axis tissues (*p* < 0.05).

Based on the results from this study, it was suggested that these three genes indirectly took part in the reproductive start at stage III before the sexual maturity of *M. amblycephala* and may participate in reproductive regulation by the HPG axis during the process of the reproductive cycle. In addition, the leptin cooperated with leptinR to complete the gonadal development and reproduction. Moreover, as the expression levels of these genes in male were fluctuate and difficult to find a change rule, it was speculated that they may mainly control the development of ovary in *M. amblycephala*. Further studies are needed to fully elucidate the function of these genes in fishes and to better understand the crucial interplay between the expression of leptin, leptinR and leprotl1 or some impact factors in gene expression.

### 3.4. Correlation Analysis of Three Genes

According to the results of correlation analysis of three genes in the relative tissues of HPG axis at different developmental stages of gonad, we found that these genes had significant positive and fairly strict correlations (*p* < 0.05, *r* > 0.7) in the regulation of gonadal development. However, in the previous studies, a positive relationship between *leptin* and *leptinR* mRNA levels in interaction from humans to fish species had been reported [24]. Theoretically, leptin was thought to be in a positive relationship with leptinR, due to the auxo-action of appetite, body weight regulation and metabolism [41]. The leptinR may participate in stimulation on body protein synthesis.

Unfortunately, few studies examined the relationship between leptin and leprotl or leptinR and leprotl. Nevertheless, some studies about the relationship between leprotl1 and some genes or hormones might further help to understand the complex relationships among these genes. An inverse relationship between growth hormone (GH) and leprotl1 had been reported in mice, which indicated that leprotl1 influenced the liver GH signaling [40]. The related relationships and functions during the gonadal development of these three genes in fishes need be further studied.

## 4. Experimental Section

### 4.1. Experimental Animals

Healthy blunt snout bream used in this study were collected from a culture farm in Tuanfeng, Huanggang, Hubei Province, China. The experimental fish were acclimatized in the laboratory in the College of Fisheries, Huazhong Agricultural University for two weeks with water temperature being about 28 °C. Fish were fed with a commercial pelleted feed twice a day. For the gene expression analysis, six individuals from each gonadal development stage (stage I to VI for ovary and testis) were anesthetized by 100 mg·L^−1^ MS-222 (Sigma, St. Louis, MO, USA) (*n* = 6). From the development stage I to III, four tissues were sampled, including liver, ovary, testis and brain (difficult to separate the hypothalamus and pituitary at those stages); and five tissues were collected from stages IV to VI, which included hypothalamus, pituitary, liver, ovary, and testis. All sampled tissues were preserved at −80 °C after frozen in liquid nitrogen overnight. The sex of the fish and gonadal developmental stages were identified by the contour of gonad and the observed results of gonad tissue slice [23,42]. Briefly, for the female *M. amblycephala*, the oocyte was surrounded by a few squamous follicle cells and had a large nucleus surrounded by a thin layer of cytoplasm (stage I, the nucleus 2.5–10.0 μm; cell size 3.6–16.0 μm); concomitant with oocyte growth, the nucleus increased in size and the cytoplasm stained uniformly (stage II); the stage III was characterized by the appearance of yolk vesicles in the cytoplasm; the yolk vesicles increased in size and number to form several peripheral rows and give rise to cortical alveoli, the radiation belt shaped in the intercellular substance (stage IV); ovary at stage V was full of cyto-architecture vacuoles; ovulation resulted in ruptured empty or postovulatory follicles, new postovulatory follicles were readily identifiable, but they rapidly degenerated (stage VI). For the male *M. amblycephala*, testis contained spermatogonial stem cells associated with sertoli cells (stage I); spermatocyst with primary or secondary spermatocyte appeared and the appearance of spermatocytes indicated that meiosis has initiated (stage II); the lobule diameter increased and spermatocyst with spermatids appeared (stage III); all stages of developing germ cells may be present in testis, but germ cells in the same spermatogenic cysts showed the synchronous development (stage IV); large number of sperm were in the seminiferous tubules (stage V); testis declined to stage III after spermiation (stage VI).

“Guidelines for Experimental Animals” of the Ministry of Science and Technology (Beijing, China) was compiled during the experiments. Institutional Animal Care and Use Ethics Committee of Huazhong Agricultural University had approved our study. All efforts were made to minimize suffering of sampled fish species.

### 4.2. Molecular Cloning

The tissues were homogenized by grinding apparatus to extract total RNA. The total RNA were respectively dissolved in 50 µL RNase-free water after the extraction using RNAiso reagent (TaKaRa, Shuzo, Japan) according to the manufacturer’s instructions. Total RNA quality was checked with the agarose gel and concentration were measured by NanoDrop 2000 (Thermo Scientific, Waltham, MA, USA). The PrimeScript RT reagent Kit with gDNA Eraser (TaKaRa, Shuzo, Japan) was used to reverse-transcribe the first strand cDNA from the total RNA. The partial cDNA sequences of *M. amblycephala leptin*, *leptinR* and *leprotl1* were obtained from *M. amblycephala* transcriptome database [43]. The 5′-/3′- Full RACE kit (TaKaRa, Shuzo, Japan) was used to amplify the 5′ and 3′ end sequences of these genes cDNA (special amplified primers were shown in Table 3). The 5′-RACE and 3′-RACE products were ligated into pGEM-T Easy vector (Promega, Madison, WI, USA) for sequencing. After compared the cDNA sequences of *M. amblycephala* with the whole genomic sequence of *D. rerio* on National Center for Biotechnology Information (NCBI) [44], the degenerate primers of *leptin*, *leptinR* and *leprotl1* were designed for amplifying the introns. The full-length cDNA sequences and genomic sequences of these three genes were assembled by the SeqMan software (DNASTAR Inc., Madison, WI, USA).

**Table 3:** The primer sequences for genes cloning and expression in the study.

Target Gene	Primer Name	Primer Sequence (5’–3’)
**Internal Control Primers for qRT-PCR**		
β-*actin* primers	β-*actin*-F	CGGACAGGTCATCACCATTG
	β-*actin*-R	CGCAAGACTCCATACCCAAGA
18S primers	18S-F	CGGAGGTTCGAAGACGATCA
	18S-R	GGGTCGGCATCGTTTACG
**Quantitative Primers of Three Genes**		
*leptin* primers	*leptin*-F	CAGTTGAGATTGCGAGTGCC
	*leptin*-R	GTTGGAGGTAACGGGGAAGG
*leptinR* primers	*leptinR*-F	TAGACGAACCAGGGTTTGATA
	*leptinR*-R	ATTCTTGCTCTGGCAGGTAA
*leprotl1* primers	*leprotl1*-F	CAGTTGGCAGCAGTGGTGAAG
	*leprotl1*-R	CATCTATCAATGGGCGGCAGT
**Gene Specific Primers for RACE**		
*leptin* specific primers for 3’ RACE	3’-1	TTTTCCCTCTCAATGGCAGCCCTGGG
	3’-2	GTACCTGGAAAAGCTTTGCCTGAA
*leptin* specific primers for 5’ RACE	5’-1	CACATCAACACACCAGAGAAAGTC
	5’-2	CCAACATACTACCAGCTTC
*leptinR* specific primers for 3’ RACE	3’-1	TTTTCCCTCTCAATGGCAGCCCTGG
	3’-2	GGTACCTGGAAAAGCTTTGCCTGAA
*leptinR* specific primers for 5’ RACE	5’-1	CACATCAACACACCAGAGAAAG
	5’-2	TCCCAACATACTACCAGCTTC
*leprotl1* specific primers for 3’ RACE	3’-1	GCATTGTGAGGTTTCCAGATTTCCCA
	3’-2	ATCCCCCCGAAACTAATGAAGAAGCAG
*leprotl1* specific primers for 5’ RACE	5’-1	GTGTGTTTATGAATCTACGCA
	5’-2	CGAAAATGGCATTTATTTAAAAAAAGAAC
**Introns Primers for Genomic Sequence**		
*leptin*-Intron	F-primer	ATCATGGCCCGAACTACCATC
	R-primer	TGTCCATGTTCAGGCAAAGC
*leptinR*-Intron-1	F-primer	TTCATTGCCGTTTCACAAGG
	R-primer	AGGGCAAGTGATTTACTAGAGG
*leptinR*-Intron-2	F-primer	TTGTGAGCTGCCCTTTGCTC
	R-primer	ATCTTCGGTAACAGTGCGGT
*leptinR*-Intron-3	F-primer	ATCTGCGAATGGGACTACCG
	R-primer	TTCAGAGGCACCCATGAACG
*leptinR*-Intron-4	F-primer	AGGTCAGAGGTCGTTCATGG
	R-primer	AGCTTCTTCAGTGCTCTCCG
*leptinR*-Intron-5	F-primer	GTGCGAGTAAAGCTGTGTGG
	R-primer	TGCCTTCTGTGATTGAGCACT
*leptinR*-Intron-6	F-primer	TGGAACCACAGGCAGAGATT
	R-primer	CACTGACAGGAACGCAATGAT
*leptinR*-Intron-7	F-primer	ATTGCGTTCCTGTCAGTGGT
	R-primer	GAAGTCCATTCCTTTTGCCCAG
*leptinR*-Intron-8	F-primer	CCTGGGCAAAAGGAATGGAC
	R-primer	CTGGAATGGCGAGGATTGGT
*leprotl1*-Intron-1	F-primer	TCGCGAGGAGTTTCTTTAGCTT
	R-primer	TATAAACGGGCAGAGCGCAG
*leprotl1*-Intron-2	F-primer	AGTCTGTCGTTTGGAGGAGC
	R-primer	CAATCCCCGTGGTCAGGAAT
*leprotl1*-Intron-3	F-primer	ACTCGGCTAGTAATGCCTGC
	R-primer	TGAAGACGGATGAGACGCTG

### 4.3. Sequence Analysis

After genes assemble in *M. amblycephala*, the putative amino acid sequences of *leptin*, *leptinR* and *leprotl1* were predicted by Open Reading Frame Finder on the NCBI [44]. We searched homologous sequences of *leptin*, *leptinR* and *leprotl1* cDNA in GenBank using the searching tool Blastn from NCBI website and predicted amino acid sequences of these three genes analyzed by DNAstar software (Madison, WI, USA). For the presence of signal peptides, SignalP 4.0 Server [45] was used to analyze the putative amino acid sequences. Besides, based on the UniProt [46] and SMART [47] database, the protein domains were marked and ExPASy online tools [48] were used to perform their analysis. TMHMM [49] were used to detect the transmembrane domains. MEGA 5.0 with the neighbor-joining method was used to construct the phylogenetic analysis of the putative amino acid sequences of *leptin*, *leptinR* and *leprotl1*. The bootstrap method with 1000 pseudo-replications was conducted to evaluate the reliability of the estimated tree.

### 4.4. Quantitative Real-Time PCR Analysis of Tissue Expression

Expression patterns of the three genes (*leptin*, *leptinR* and *leprotl1*) were analyzed basing on quantitative real-time PCR (qRT-PCR) and using the cDNA of various tissues in different stages of *M. amblycephala* gonad as templates. The *β-actin* and *18S* rRNA were selected as two reference genes based on their expression stability, and all primer sequences were described in Table 3. The qRT-PCR assay was performed using SYBR Premix Ex Taq™ (TaKaRa, Dalian, China) on a Roche Light Cycler 480 machine (Roche, Sussex, UK). The qRT-PCR conditions were as follows: denaturation at 95 °C for 30 s, followed by 40 cycles of 95 °C for 5 s, annealing at 58 °C (*β-actin*)/55 °C (*leptin*)/58 °C (*leptinR*)/57.3 °C (*leprotl1*) for 20 s, and elongation at 72 °C for 15 s. The relative quantification of the target and reference genes was evaluated according to standard curves. Each experiment was conducted in triplicate. The relative stability measure (M) of the reference genes was calculated by GeNorm [50] as described in our previous studies was used to select the reference genes with the most stable expression [51]. According to the results, the *β-actin* was chosen as the reference gene in following analysis for its more stable than 18S rRNA.

### 4.5. Statistical Analysis

For statistical analysis, data from qRT-PCR was presented as the mean ± SE. The optimized comparative Ct (2^−ΔΔ*C*t^) value method [52] was utilized to compute the relative expression value. One-way ANOVA and *t*-test were used to compare the significance of *leptin*, *leptinR* and *leprotl1* genes between males and females as well as different gonadal development stages. Duncan’s test was applied to multiple comparisons by IBM SPSS Statistics 19.0 (SPSS, Chicago, IL, USA). Differences were considered significant at *p* < 0.05 and greatly significant at *p* < 0.01.

## 5. Conclusions

In conclusion, this study firstly reported the relationship between *leptin*, *leptinR* and *leprotl1*, as well as the differential expression patterns of these tree genes in *M. amblycephala*. Additionally, our results had revealed that the expressions of *leptin*, *leptinR* and *leprotl1* were correlated with each other both in related tissues of HPG axis during the different developmental stages of gonad. These results could be useful for further investigation about the regulation mechanism of *leptin*, *leptinR* and *leprotl1* genes in fish reproduction system.

## Supplementary Materials

Supplementary materials can be found at http://www.mdpi.com/1422-0067/16/11/26044/s1.
