# Supplementary material for: Leptin Genes in Blunt Snout Bream: Cloning, Phylogeny and Expression Correlated to Gonads Development
Source: Int J Mol Sci. 2015 Nov 18;16(11):27609–24. doi: 10.3390/ijms161126044 (PMC4661902; doi:10.3390/ijms161126044)
Supplement: Supplementary file 1 [file ijms-16-26044-s001.pdf]

Supplementary Materials: *Leptin* Genes in Blunt Snout Bream: Cloning, Phylogeny and Expression Correlated to Gonads Development

Honghao Zhao, Cong Zeng, Shaokui Yi, Shiming Wan, Boxiang Chen and Zexia Gao

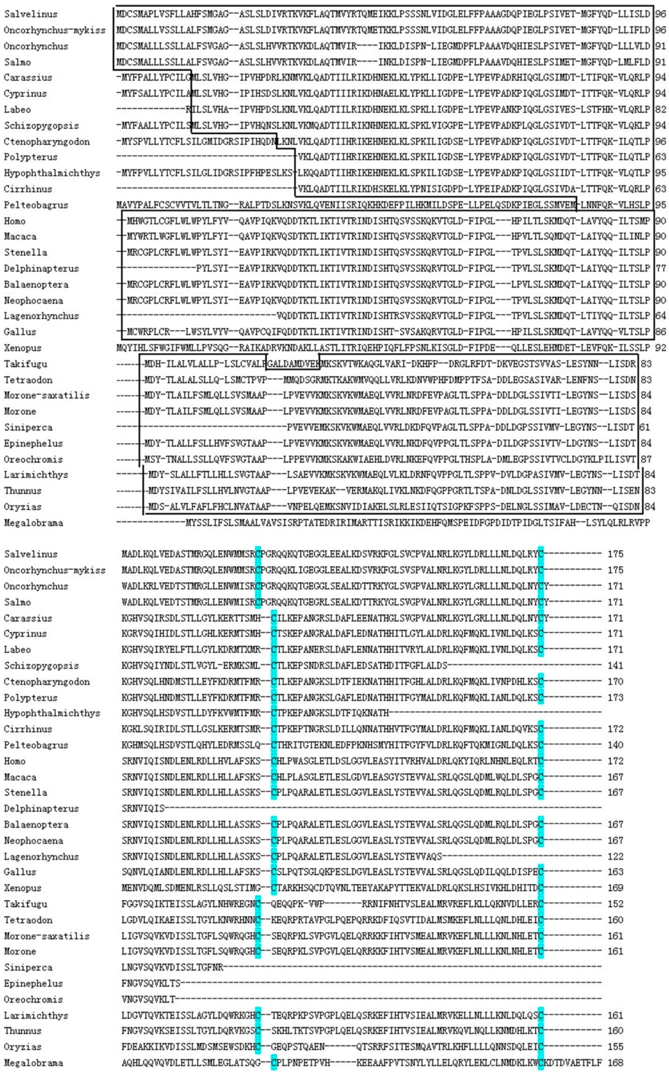

Figure S1. Molecular characterization of vertebrate *leptin*. Comparison of amino acid sequences of the teleosts, amphibians, birds, mammalias and human *leptin*. Multiple sequences alignment was performed by Clustal X. Conserved cysteine and aa. residues involved in the formation were shaded. The highly conserved amino acid sequences between various vertebrates were boxed. The conservative disulfide bridge were colored in blue. GenBank Accession Nos.: *Homo sapiens* (NP_000221), *Macaca mulatta* (NP_001036220), *Lagenorhynchus albirostris* (ABK88255), *Balaenoptera acutorostrata* (AER93242), *Neophocaena phocaenoides asiaeorientalis* (AER93239), *Stenella attenuata* (AER93241), *Delphinapterus leucas* (AGT57736), *Gallus gallus* (AAC60368), *Xenopus laevis* (NP_001089183), *Carassius auratus* (ACL68083), *Cyprinus carpio "jian"* (AGK24955), *Labeo rohita* (ADB22376), *Cirrhinus molitorella* (ADI70505), *Schizopygopsis pylzovi* (AFY23973), *Ctenopharyngodon idella* (ACI32423), *Polypterus senegalus* (ADI70506), *Hypophthalmichthys molitrix* (ACI32424), *Pelteobagrus fulvidraco* (AFO67938), *Salvelinus alpinus* (BAH83535), *Oncorhynchus mykiss* (AGB34181), *Oncorhynchus keta* (AGU36310), *Salmo salar* (ACZ02412), *Takifugu rubripes* (NP_001027897), *Tetraodon nigroviridis* (BAD94451), *Oryzias latipes* (BAD94448), *Thunnus thynnus* (ADT91717), *Morone saxatilis* (AFD34357), *Morone chrysops* (AFD34356), *Epinephelus coioides* (BAI66433), *Oreochromis niloticus* (AER12722), *Siniperca chuatsi* (ACT98260), *Larimichthys crocea* (AGR51148).

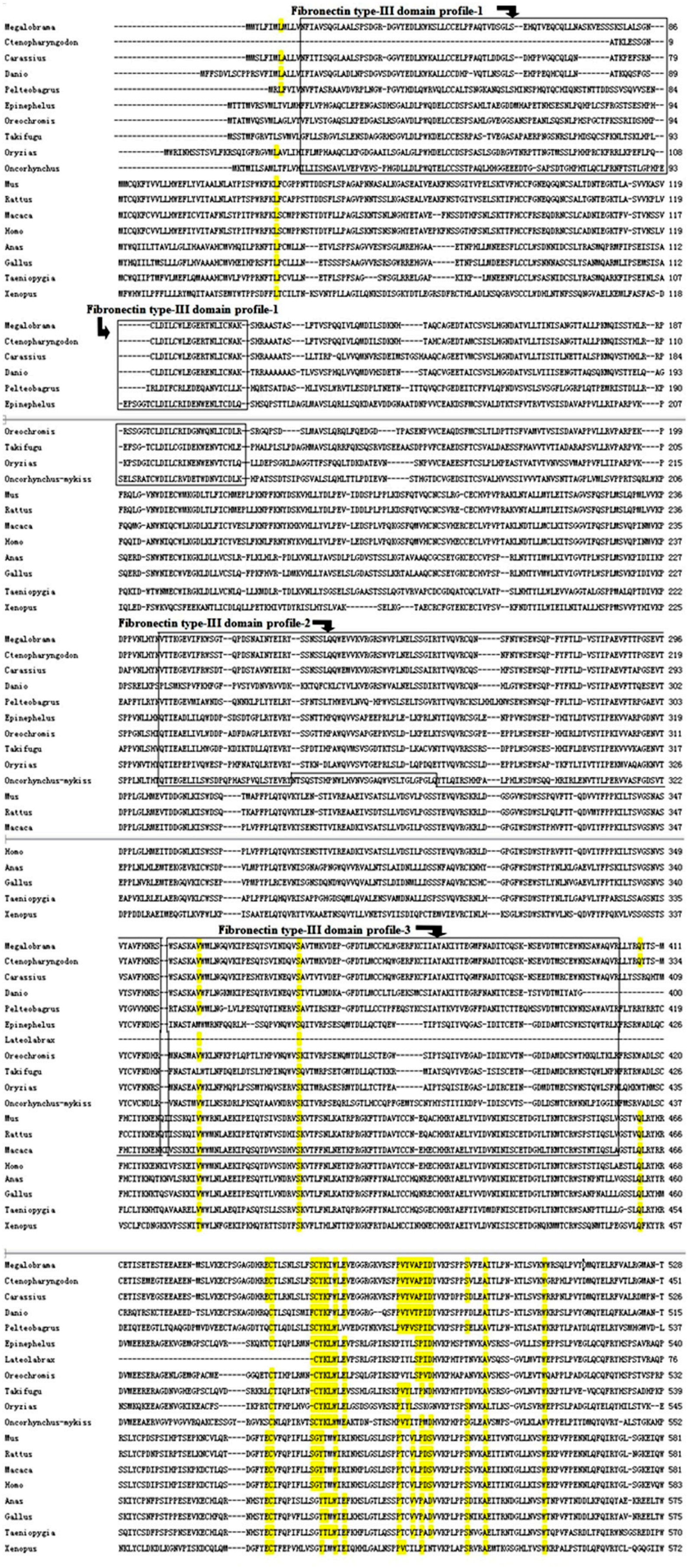

Figure 2. *Cont.*

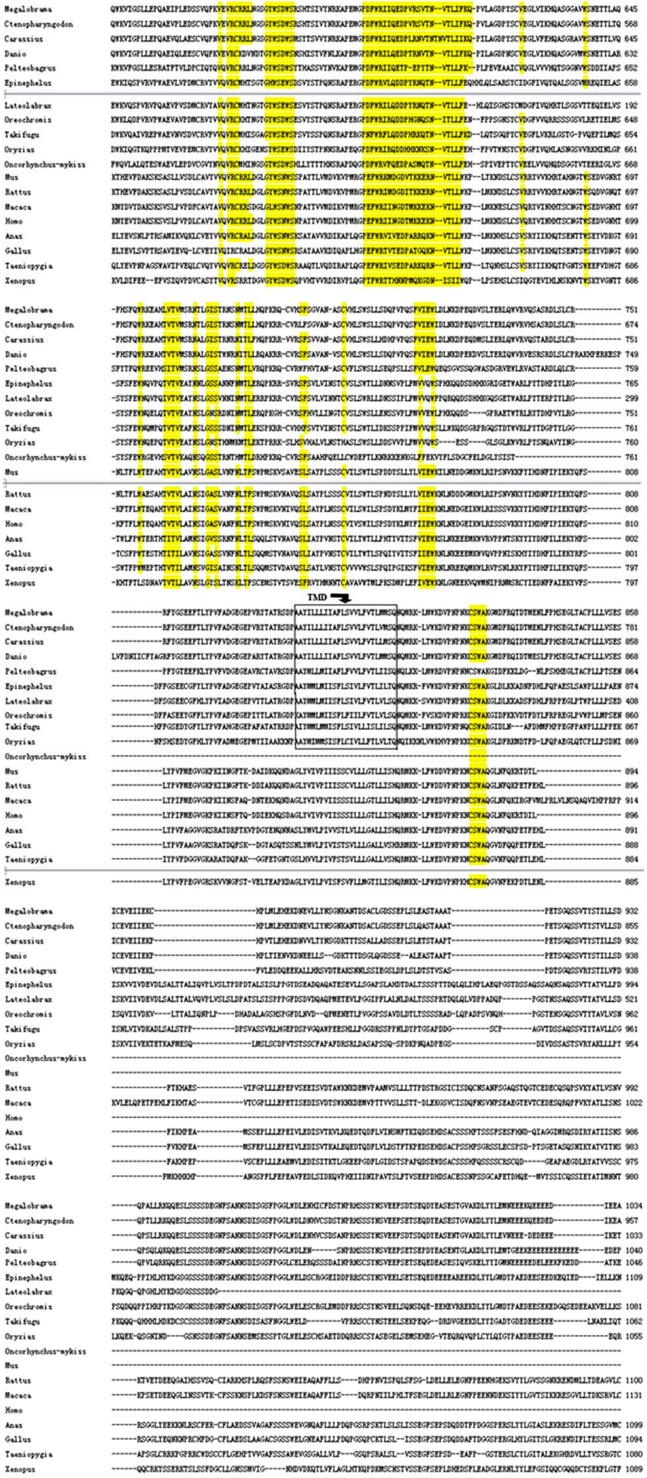

Figure S2. Molecular characterization of vertebrate *leptinR*. Comparison of amino acid sequences of the teleosts, amphibians, birds, mammalias and human *leptinR*. Multiple sequences alignment was performed by Clustal X. Conserved cysteine and aa. residues involved in the formation were shaded. The highly conserved functional domain amino acid sequences between various vertebrates were boxed and highlighted in yellow. GenBank Accession Nos.: *Carassius carassius* (ADZ75460), *Ctenopharyngodon idella* (AFU35431), *Danio rerio* (NP_001106847), *Epinephelus coioides* (AFU55262), *Lateolabrax japonicus* (AHI85769), *Oncorhynchus-mykiss* (AGO59893), *Oreochromis mossambicus* (AGT28753), *Oryzias melastigma* (ABC86922), *Pelteobagrus fulvidraco* (AFO67946), *Takifugu rubripes* (NP_001124341), *Xenopus* (NP_001037866), *Mus musculus* (AAA93014), *Rattus norvegicus* (NP_036728), *Anas platyrhynchos* (ACF17729), *Gallus* (NP_989654), *Taeniopygia guttata* (AFK25169), *Macaca mulatta* (NP_001027991), *Homo sapiens* (AAC23650).

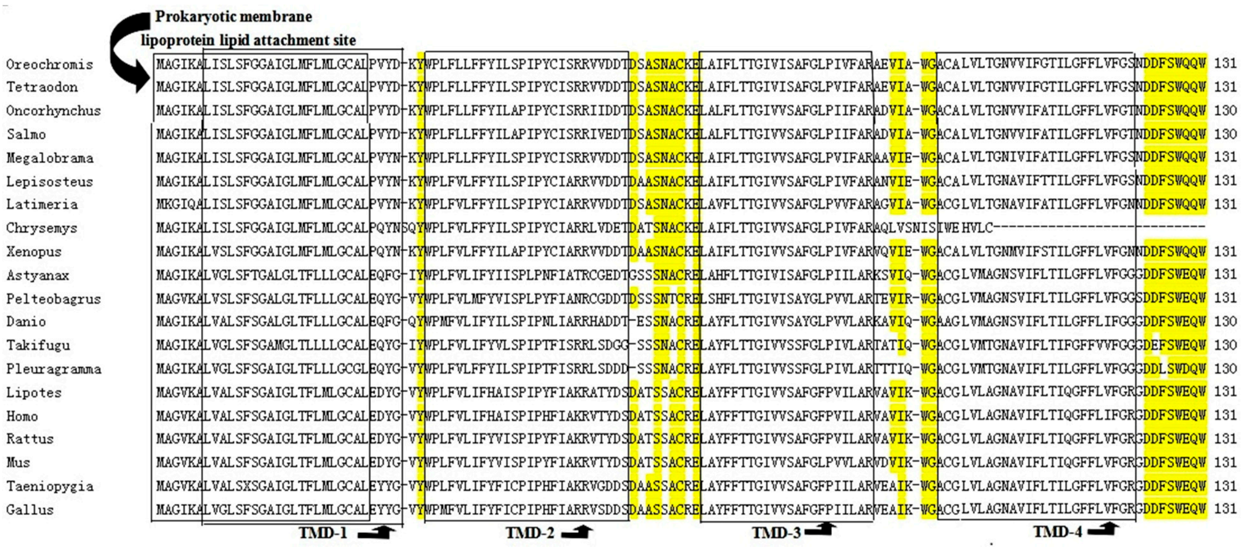

Figure S3. Molecular characterization of vertebrate *leprotl1*. Comparison of amino acid sequences of the teleosts, amphibians, birds, mammalias and human *leprotl1*. Multiple sequences alignment was performed by Clustal X. Conserved cysteine and aa.residues involved in the formation were shaded. The highly conserved functional domain amino acid sequences between various vertebrates were boxed and marked in yellow. GenBank Accession Nos.: *Homo sapiens* (AAH56250), *Lipotes vexillifer* (XP_007460146), *Rattus norvegicus* (AAH62003), *Mus musculus* (AAH04744), *Gallus* (NP_001007959), *Taeniopygia guttata* (XP_004174372), *Takifugu rubripes* (NP_001129621), *Pleuragramma antarctica* (AES12469), *Danio rerio* (NP_001017787), *Astyanax mexicanus* (XP_007250701), *Pelteobagrus fulvidraco* (AFN08752), *Chrysemys picta bellii* (XP_005308635), *Lepisosteus oculatus* (XP_006629222), *Latimeria chalumnae* (XP_006010007), *Oreochromis niloticus* (XP_003443415), *Tetraodon nigroviridis* (CAG04698), *Oncorhynchus mykiss* (ACO07979), *Salmo salar* (NP_001139931), *Xenopus laevis* (NP_001079518).

Table S1. The expression levels of *M. amblycephala leptin, leptinR* and *leprotl1* in the different tissues during the developmental periods of gonads (mean ± SE). The β-actin was used as an internal control to calibrate the cDNA template for all the samples. Values with one asterisk mean significantly different between females and males at $p < 0.05$ and two asterisks at $p < 0.01$.

Stage	Tissues	***Leptin***		***LeptinR***		***Leprotl1***	
		♀	♂	♀	♂	♀	♂
I	Gonad	0.00004 ± 0.00000	0.00069 ± 0.00011 **	0.00024 ± 0.00000	0.00791 ± 0.00004 **	0.29260 ± 0.01450 **	0.01232 ± 0.00084
	Brain	0.00002 ± 0.00000	0.00002 ± 0.00000	0.00256 ± 0.00104 **	0.00146 ± 0.00029	0.13472 ± 0.01431	0.13513 ± 0.00572
	Liver	0.00227 ± 0.00220 **	0.00012 ± 0.00001	0.01685 ± 0.00054 **	0.00160 ± 0.00028	0.03017 ± 0.00806	0.03661 ± 0.00498
II	Gonad	0.00002 ± 0.00000 *	0.00001 ± 0.00000	0.00007 ± 0.00000	0.00076 ± 0.00005 **	0.19489 ± 0.00321 **	0.10926 ± 0.00355
	Brain	0.00538 ± 0.00075	0.01761 ± 0.00097 **	0.00256 ± 0.00104	0.01409 ± 0.00248 **	0.15579 ± 0.01780	0.23520 ± 0.02510
	Liver	0.33466 ± 0.03921 **	0.00517 ± 0.00047	0.17830 ± 0.02288 **	0.00806 ± 0.00054	0.18250 ± 0.02898 **	0.01364 ± 0.00070
III	Gonad	0.00002 ± 0.00000	0.00071 ± 0.00010 **	0.00026 ± 0.00001	0.00230 ± 0.00009 **	0.15500 ± 0.00076 **	0.08733 ± 0.00719
	Brain	0.75896 ± 0.10136 **	0.01881 ± 0.00056	0.66882 ± 0.06486 **	0.01973 ± 0.00134	9.47115 ± 0.16127 **	0.69158 ± 0.01815
	Liver	0.01416 ± 0.00204 *	0.02808 ± 0.00425	0.01651 ± 0.00442	0.01898 ± 0.00090	0.02690 ± 0.00569	0.03719 ± 0.00389
IV	Gonad	0.00000 ± 0.00000	0.00017 ± 0.00001 **	0.00007 ± 0.00001	0.00353 ± 0.00043 **	0.13534 ± 0.00236 **	0.08337 ± 0.00510
	Liver	0.03303 ± 0.00398	0.07071 ± 0.00786 **	0.02655 ± 0.00204	0.02934 ± 0.00869	0.02301 ± 0.00258	0.03986 ± 0.00793
	Pituitary	0.00005 ± 0.00001	0.00238 ± 0.00026 **	0.00327 ± 0.00035	0.00644 ± 0.00048 **	0.05043 ± 0.01079	0.06002 ± 0.00653
	Hypothalamus	0.00002 ± 0.00000	0.00039 ± 0.00008 **	0.00175 ± 0.00011	0.00177 ± 0.00068	0.06132 ± 0.00132 **	0.03374 ± 0.00575
V	Gonad	0.00002 ± 0.00000 *	0.00001 ± 0.00000	0.00238 ± 0.00027 **	0.00023 ± 0.00002	0.08383 ± 0.00347	0.07955 ± 0.00549
	Liver	0.05244 ± 0.00928	0.05547 ± 0.00677	0.04631 ± 0.00957	0.02909 ± 0.01057	0.04150 ± 0.00836 **	0.01111 ± 0.00142
	Pituitary	0.27409 ± 0.01427	0.30982 ± 0.02556	0.17942 ± 0.02390 **	0.00434 ± 0.00157	2.45742 ± 0.44028 **	0.03102 ± 0.00809
	Hypothalamus	0.00109 ± 0.00011	0.00400 ± 0.00068 **	0.00364 ± 0.00047	0.32645 ± 0.05979 **	0.02967 ± 0.00426	0.08459 ± 0.00440 **
VI	Gonad	0.00730 ± 0.00028	0.01750 ± 0.00134 **	0.00790 ± 0.00093	0.01465 ± 0.00038 **	0.04953 ± 0.00673	0.15342 ± 0.01387 **
	Liver	0.07953 ± 0.00533	0.35823 ± 0.02554 **	0.06165 ± 0.00407	0.08643 ± 0.01226	0.03885 ± 0.00275	0.03308 ± 0.01460
	Pituitary	0.26661 ± 0.01539 *	0.17994 ± 0.01491	0.18502 ± 0.02369 **	0.00378 ± 0.00053	0.10973 ± 0.04010	0.13869 ± 0.04312
	Hypothalamus	0.00004 ± 0.00000	0.00733 ± 0.00303 **	0.00356 ± 0.00008	0.22562 ± 0.05814 **	0.03374 ± 0.00574	0.03374 ± 0.00575
